# Mapping Brain Microstructure and Network Alterations in Depressive Patients with Suicide Attempts Using Generalized Q-Sampling MRI

**DOI:** 10.3390/jpm11030174

**Published:** 2021-03-03

**Authors:** Vincent Chin-Hung Chen, Chun-Ju Kao, Yuan-Hsiung Tsai, Roger S. McIntyre, Jun-Cheng Weng

**Affiliations:** 1 School of Medicine, Chang Gung University, Taoyuan 33302, Taiwan; cch1966@gmail.com (V.C.-H.C.); russell.tsai@gmail.com (Y.-H.T.); 2 Department of Psychiatry, Chang Gung Memorial Hospital, Chiayi 61363, Taiwan; 3 Department of Medical Imaging and Radiological Sciences, Bachelor Program in Artificial Intelligence, Chang Gung University, No. 259, Wenhua 1st Rd., Taoyuan 33302, Taiwan; jameskao8673@gmail.com; 4 Department of Diagnostic Radiology, Chang Gung Memorial Hospital, Chiayi 61363, Taiwan; 5 Mood Disorder Psychopharmacology Unit, University Health Network, Department of Psychiatry, University of Toronto, Toronto, ON M5S, Canada; Roger.McIntyre@uhn.ca; 6 Institute of Medical Science, University of Toronto, Toronto, ON M5S, Canada; 7 Departments of Psychiatry and Pharmacology, University of Toronto, Toronto, ON M5S, Canada; 8 Medical Imaging Research Center, Institute for Radiological Research, Chang Gung University and Chang Gung Memorial Hospital at Linkou, Taoyuan 33302, Taiwan

**Keywords:** suicide attempt, generalized q-sampling imaging, graph theoretical analysis, network-based statistical analysis

## Abstract

Depressive disorder is one of the leading causes of disability worldwide, with a high prevalence and chronic course. Depressive disorder carries an increased risk of suicide. Alterations in brain structure and networks may play an important role in suicidality among depressed patients. Diffusion magnetic resonance imaging (MRI) is a noninvasive method to map white-matter fiber orientations and provide quantitative parameters. This study investigated the neurological structural differences and network alterations in depressed patients with suicide attempts by using generalized q-sampling imaging (GQI). Our study recruited 155 participants and assigned them into three groups: 44 depressed patients with a history of suicide attempts (SA), 56 depressed patients without a history of suicide attempts (D) and 55 healthy controls (HC). We used the GQI to analyze the generalized fractional anisotropy (GFA) and normalized quantitative anisotropy (NQA) values in voxel-based statistical analysis, topological parameters in graph theoretical analysis and subnetwork connectivity in network-based statistical analysis. GFA indicates the measurement of neural anisotropy and represents white-matter integrity; NQA indicates the amount of anisotropic spins that diffuse along fiber orientations and represents white-matter compactness. In the voxel-based statistical analysis, we found lower GFA and NQA values in the SA group than in the D and HC groups and lower GFA and NQA values in the D group than in the HC group. In the graph theoretical analysis, the SA group demonstrated higher local segregation and lower global integration among the three groups. In the network-based statistical analysis, the SA group showed stronger subnetwork connections in the frontal and parietal lobes, and the D group showed stronger subnetwork connections in the parietal lobe than the HC group. Alternations were found in the structural differences and network measurements in healthy controls and depressed patients with and without a history of suicide attempt.

## 1. Introduction

More than 260 million people are affected by various degrees of depression [1]. Depressive disorder is one of the dominant causes of disability and mortality worldwide and is also highly prevalent and recurrent [2,3]. People with depression will probably encounter deficiencies in emotion processing, memory and executive function [4], which may lead to critical inconveniences in their lives. Depression can also be triggered by numerous kinds of factors, including aging, childhood maltreatment, psychological stress, chronic medical illnesses and so on [5,6,7]. A previous study showed that major depressive disorder carries an increased risk of suicide [8]. Suicide has continuously been a serious public health issue. More than 800,000 people die by suicide every year throughout the world; in addition, suicide is one of the top leading causes of death, especially for younger generations [9]. Previous research also shown that the risk of attempting suicide is critical [10].

In previous studies, researchers mainly focused on biological differences and physical behaviors instead of brain network differences [11,12]. However, network alterations in the brain are important factors for patients with depression as well as suicidal ideation and may be linked to the risk of suicide attempts [13]. In recent years, an increasing number of neuroimaging studies have shown that functional and structural changes in the brain may be related to depression and suicidal behaviors. Diffusion MRI is a noninvasive method to map white-matter fiber orientations and provide quantitative parameters and has been widely used for neuroscience research, especially for psychiatric disorders. Diffusion tensor imaging (DTI) is specifically used to characterize gross fiber orientations. Previous study used DTI for research on white matter integrity to evaluate the value of fractional anisotropy (FA) in patients with suicidal ideation, and FA is the mensuration of neural anisotropy [14]; however, DTI is not able to distinguish branching or crossing fibers [15]. Therefore, we adopted a different method with a unique reconstruction approach called generalized q-sampling imaging (GQI), which can be applied to an extensive q-space dataset to improve the accuracy of fiber tracking and extract quantitative and directional information about complex fibers as well. Previous studies also showed that the GQI shows good specificity and sensitivity for the evaluation of white matter integrity [15].

Graph theory is an analysis of graphs that illustrates the connections of objects by defining pairwise interconnections called “edges” and “nodes” [16]. In this study, edges represent the functional or structural connections between regions, and nodes represent different brain regions. The brain is a complicated organ with both integrated and segregated functional characteristics [17]. Therefore, graph theoretical approaches can be constructed to explore network changes in the brain and obtain further perspectives of brain connectivity. The network of white matter that indicates the connection of neural fibers between brain regions and the structural network of the brain is deemed to have a characteristic of “small world” property that indicates the brain is equipped with the abilities of global integration and local segregation [18]. A deficiency in emotion processing could lead to structural changes in the brain. The graph theoretical approach can be a method to investigate brain network disturbances. It has also been used to explore Alzheimer’s disease, schizophrenia, and other traumatic brain injuries [19,20,21]. A previous DTI study found that depression is associated with distributed brain networks by network controllability analysis; however, the chosen brain modularity partition might bias the result [22]. Therefore, our study performed graph theoretical analysis to investigate network alternations.

Our study aimed to compare the neurologically structural differences and network alterations among three groups of people, including healthy controls (HCs), depressed patients with a history of suicide attempts (SA) and depressed patients without a history of suicide attempts (D), by using comprehensive GQI and graph theoretical analysis.

## 2. Materials and Methods

### 2.1. Participants

In our cross-sectional study, a total of 155 participants were recruited from the Department of Psychiatry at Chiayi Chang Gung Hospital and assigned into three groups: 44 depressed patients with a history of suicide attempts (SA), 56 depressed patients without a history of suicide attempts (D) and 55 healthy controls (HC). The assessments of patients confirmed to have depressive disorder, suicidal ideation as well as suicide attempt were based on the diagnoses of psychiatrists, the structural interview of the Mini-International Neuropsychiatric Interview (MINI) and the Beck suicide intent scale (administered by the research nurse). The inclusion criteria for all participants were as follows: aged over 20 years old and right-handed. The exclusion criteria for all participants were pregnancy, history of severe brain damage, previous diagnosis with other mental disorders, illiteracy and MRI contraindications. If participants feel any discomfort during the scanning process, the scanning will stop, and he or she will be excluded from the experiment. Adequate communication was performed to ensure that all participants were emotionally stable and had clearly understood the experimental procedure and relevant guidelines. Our study was approved by the Institutional Review Board of the Chang Gung Memorial Hospital, Chiayi, Taiwan.

### 2.2. Diffusion MRI Data Acquisition

All 155 participants were scanned by a 3 T MRI imaging system (Verio, SIEMENS, Germany) at Chiayi Chang Gung Memorial Hospital. For diffusion imaging, a single-shot, diffusion-weighted spin echo-planar imaging sequence was performed. To reduce motion and scanner noise, cushions and earmuffs were provided for participants. The image acquisition parameters were as follows: repetition time (TR)/echo time (TE) = 8943/115 msec, number of excitations = 1, field of view (FOV) = 250 × 250 mm^2^, slice thickness = 4 mm, matrix size = 128 × 128, voxel size = 3.4 × 3.4 × 4 mm^3^, *b*-values = 0, 1000, 1500 and 2000 s/mm^2^ in 193 total noncollinear directions. The total acquisition time of each participant was approximately 30 min.

### 2.3. Generalized Q-Sampling Imaging (GQI)

The GQI contains several quantitative indices that can provide additional information, including generalized fractional anisotropy (GFA), quantitative anisotropy (QA), normalized quantitative anisotropy (NQA) and the isotropic value of the orientation distribution function (ISO) [15]. GFA is introduced for the analysis of white matter diffusion properties and indicates the measurement of neural anisotropy; QA represents the amount of anisotropic spins that diffuse along fiber orientations; NQA is the normalized QA; ISO is defined as the background isotropic diffusion. QA and ISO represent the diffusion of water in a restricted direction and in an isotropic fashion, respectively [15,23]. QA and ISO represent how much water diffuses (i.e., density) in a specific/restricted direction and in an isotropic fashion (i.e., total isotropic component), respectively. In contrast, GFA, which is calculated from an orientation distribution function, is a measure of how fast water diffuses (i.e., diffusivity) in an anisotropic fashion, i.e., it represents degree to which diffusion is anisotropic [23]. In this study, we particularly investigated the value of GFA and NQA. In addition, all participants’ age, gender, years of education, Hamilton Rating Scale for Depression (HAM-D) score without suicidal factors and Hospital Anxiety and Depression Scale (HADS) score (part of anxiety) were considered 5 covariates of no interest.

### 2.4. Diffusion Imaging Preprocessing

Before performing the image analysis, the quality of every image was checked and assessed. The eddy current correction by FSL (FMRIB Software Library) was performed to ensure the quality of diffusion imaging by reducing eddy current artifacts, which are caused by echo planar imaging sequences and may distort the image. We used SPM (statistical parametric mapping; Wellcome Department of Cognitive Neurology, London, UK) to normalize the sizes and shapes of all imaging data. Then, we reconstructed two indices of GQI: GFA and NQA by DSI Studio.

### 2.5. Voxel-Based Statistical Analysis

We specifically reconstructed and analyzed two GQI indices of GQI, GFA and NQA, by DSI Studio. ANCOVA was performed to observe the differences between the three groups. Then, post hoc two-sample *t*-tests were performed to assess two indices (GFA and NQA values) among brain regions for further evaluation among the three groups using SPM. A false discovery rate (FDR)-corrected *p* value of < 0.05 was considered to be statistically significant.

### 2.6. Graph Theoretical Analysis (GTA)

GQI data were utilized for graph theoretical analysis (GTA). In GTA, we defined a set of edges and nodes. We reconstructed pathways of neural fibers of the brain using DSI Studio and segmented the brain into 90 regions of interest (ROIs) in Montreal Neurological Institute (MNI) space based on the Automated Anatomical Labeling (AAL) atlas [24]. Every brain region was defined as a node [25]. Edges are the connectivity between each node. The degree of a node is the number of edges that connect to the rest of the network, which specifies the edge distribution of nodes in brain networks [26]. We can acquire topological properties of complicated brain networks by using graph theoretical analysis, including parameters that illustrate global integration (such as global efficiency, normalized shortest path length and characteristic path length) and parameters that illustrate local segregation (such as modularity and the normalized clustering coefficient) and the small-worldness index. The area under the curve (AUC) of topological parameters within selected ranges was compared between groups. The density represents the fraction of present connections to all possible connections. The network density range was calculated between 0.05 and 0.3, with an increment of 0.01. Furthermore, to identify the statistically significant difference, we manually extracted the AUC of the density between 0.05 and 0.3 to execute two-sample *t*-tests to calculate the *p* value within groups.

### 2.7. Network-Based Statistical (NBS) Analysis

We used network-based statistical (NBS) analysis to identify significant differences in any connected subnetwork in the brain and made group comparisons [27]. NBS analysis is based on a statistically parametric map with cluster-based suprathreshold links, and the structural topological extent can be applied to identify the significance. The null distribution of the edge number was experientially obtained by nonparametric permutation (5000 times) to assess the significance of connected edges. Then, BrainNet viewer (The MathWorks Inc., Natick, MA, USA) was used to identify the significant subnetworks pictured by NBS.

## 3. Results

### 3.1. Demographic Characteristics

The demographic characteristics of all participants are shown in Table 1. In addition, age, gender, years of education, Hamilton Rating Scale for Depression (HAM-D) score without suicidal factors and Hospital Anxiety and Depression Scale (HADS) score (part of anxiety) were five covariates considered to adjust the impact of each factor for subsequent analyses to investigate differences among groups.

### 3.2. Voxel-Based Statistical Analysis

**Figure 1:**
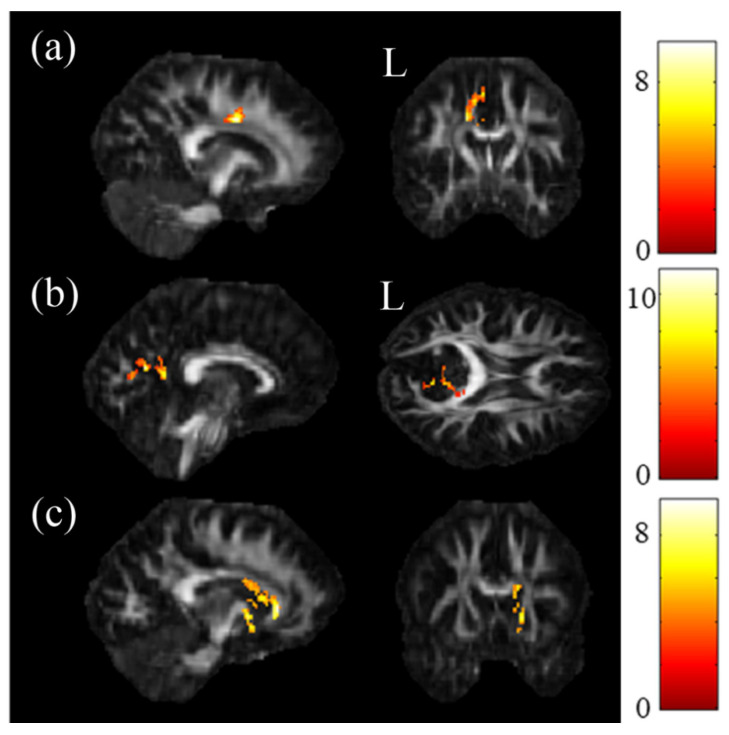
ANCOVA results of generalized fractional anisotropy (GFA) and normalized quantitative anisotropy (NQA). Significant results were demonstrated in the (**a**) cingulate gyrus and the (**b**) precuneus of GFA value and in the (**c**) cingulate gyrus and the caudate of NQA value (*p* < 0.05, cluster size > 100, color bar: F scores).

**Figure 2:**
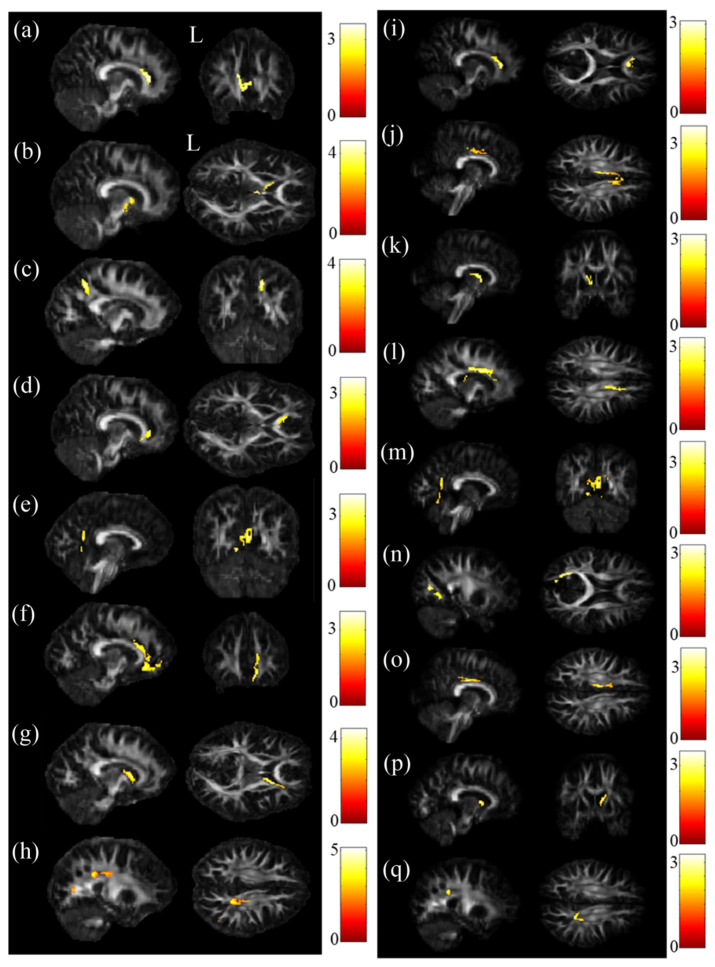
Post hoc t-test results. Compared with the HC group, significantly lower GFA values in the (**a**) corpus callosum, cingulate gyrus, (**b**) caudate and (**c**) precuneus occurred in the SA group. Compared with the D group, the SA group had significantly lower GFA values in the (**d**) corpus callosum and (**e**) precuneus. Compared with the HC group, the D group had significantly lower GFA values in the (**f**) cingulate gyrus, (**g**) caudate and (**h**) precuneus. Compared with the HC group, significantly lower NQA values in the corpus (**i**) callosum, (**j**) cingulate gyrus and (**k**) caudate occurred in the SA group. Compared with the D group, significantly lower NQA values in the (**l**) corpus callosum, (**m**) precuneus and (**n**) cuneus occurred in the SA group. Compared with the HC group, the D group had significantly lower NQA values in the (**o**) cingulate gyrus, (**p**) caudate and (**q**) precuneus (*p* < 0.05, cluster size > 100, color bar: t scores).

The ANCOVA results demonstrated that the GFA value showed significant differences in the cingulate gyrus and precuneus (Figure 1a,b), and the NQA value showed significant differences in the cingulate gyrus and caudate (Figure 1c) (*p* < 0.05). Then, post hoc two-sample *t*-tests were performed to assess GFA and NQA values among these brain regions. In two-sample t-tests, both GFA and NQA values of the corpus callosum, cingulate gyrus and caudate were significantly lower in the SA group than in the HC group (Figure 2a,b,i–k). Additionally, the precuneus had a lower GFA value in the SA group than in the HC group (Figure 2c). In the comparison between the SA group and the D group, both the corpus callosum and precuneus had notably lower GFA and NQA values in the SA group than in the D group (Figure 2d,e,l,m). Additionally, the cuneus had a lower value of NQA in the SA group than in the D group (Figure 2n). In the comparison between the D group and HC group, both GFA and NQA values of the cingulate gyrus, caudate and precuneus were remarkably lower in the D group than in the HC group (Figure 2f,g,h,o–q). Furthermore, the cuneus had a lower GFA value in the D group than in the HC group (Figure 2h).

### 3.3. Graph Theoretical Analysis

**Figure 3:**
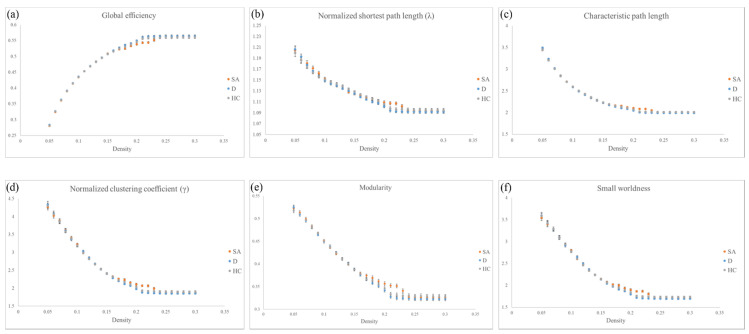
Topological parameters of graph theoretical analysis. Topological parameters including (**a**) global efficiency, (**b**) normalized shortest path length (λ), (**c**) characteristic path length, (**d**) normalized clustering coefficient (γ), (**e**) modularity and (**f**) small-worldness index among the three groups (SA, D and HC).

Individual networks of the three groups (SA, D and HC) fluctuated when the density was below 0.05, which made group comparisons meaningless. In addition, network measurements of the three groups remained unchanged when the density was above 0.3. Therefore, we assessed the density from 0.05 to 0.3, and the results were calculated by AUC analyses. Although there were no significant statistical results (*p* > 0.05) among the groups, we could still find a notable trend in some topological parameters, including global efficiency, normalized shortest path length (λ), characteristic path length, normalized clustering coefficient (γ), modularity and small worldness index. The networks of both the D and HC groups showed similar trends and had no notable differences among the six topological parameters (Figure 3a–f). However, the results of the SA group illustrated lower global efficiency as well as longer normalized shortest path length (λ) and characteristic path length than the D group and HC group (Figure 3a–c); in addition, the SA group also demonstrated a higher normalized clustering coefficient (γ) and modularity (Figure 3d,e). The small-worldness index is the ratio of the normalized clustering coefficient (γ) to the normalized shortest path length (λ). Our analyses showed that the small-worldness index of all three groups was greater than one (index values > 1).

### 3.4. Network-Based Statistical (NBS) Analysis

**Figure 4:**
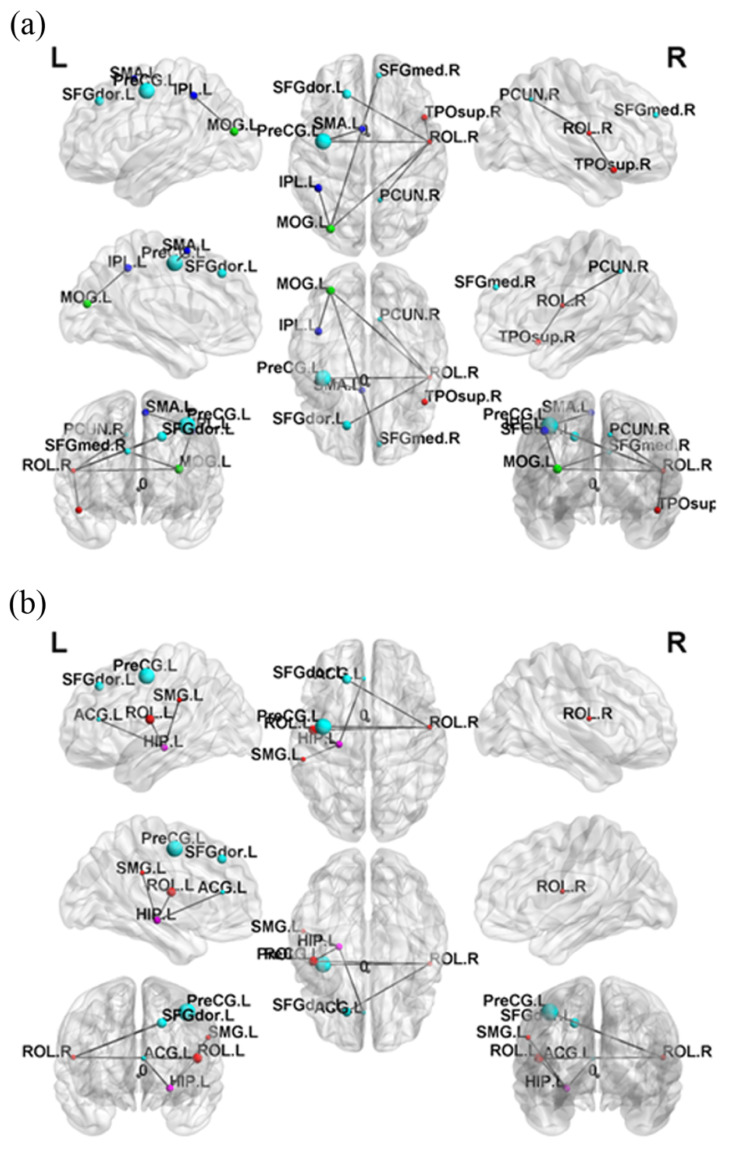
Network-based statistical analysis (NBS) results. (**a**) Compared with the HC group, the SA group demonstrated significantly stronger subnetwork connections in the frontal and parietal lobes. (**b**) Compared with the HC group, the D group demonstrated significantly stronger subnetwork connections in the parietal lobe (*p* < 0.05).

The NBS results illustrated that the SA group had stronger subnetwork connections in both the frontal and parietal lobes than the HC group (Figure 4a). Moreover, the D group demonstrated stronger subnetwork connections in the parietal lobe than the HC group (Figure 4b). In addition, no significant (*p* > 0.05) results were discovered in the comparison of subnetworks between the SA group and D group.

## 4. Discussion

In this study, we acquired images by GQI, which is more accurate than DTI methods [28]. Additionally, graph theoretical analysis is a pervasive method to explore network alternations. Our study investigated both structural and topological changes in three groups of people (healthy controls, depressed patients with and without a history of suicide attempts) and made some significant discoveries.

### 4.1. Voxel-Based Statistical Analysis

The GFA and NQA values of the three groups (SA, D and HC groups) were analyzed in our study. GFA is highly correlated with fractional anisotropy (FA) and represents the level of myelination, while NQA represents the normalized value of a measured anisotropy based on restricted diffusion [15]. In the voxel-based statistical analysis, ANCOVA and *t*-tests were used to compare groups. The ANCOVA results first demonstrated those regions that had the most significantly different values of GFA and NQA among the three groups, which are the GFA value of the cingulate gyrus and precuneus as well as the NQA value of the cingulate gyrus and caudate. These regions of the brain have been substantially highlighted to be correlated with psychiatric disorders, especially mood and anxiety disorders [29,30,31]. Furthermore, post hoc two-sample *t*-tests were compared between each group. We discovered that the corpus callosum, cingulate gyrus and precuneus showed significant results in most comparisons among the three groups, namely, the HC group had the best white matter integrity, while the SA group had the worst. The result was consistent with a previous study [32]. The neurological structural variations were primarily located in the corpus callosum, cingulate gyrus, precuneus, cuneus and caudate.

### 4.2. Corpus Callosum

Our comparison showed that the SA group had the worst white matter connectivity in the corpus callosum, while the HC group had the best connectivity. Previous studies have shown that these regions play key roles in cognitive control, working memory and emotion processing in major depressive disorder (MDD) patients [33,34,35]. The corpus callosum is also highly correlated with mood regulation [36]. Many studies have highlighted that alterations of the corpus callosum are highly associated with emotion regulation, cognitive function and executive function; therefore, with the impairment of the corpus callosum, patients easily suffer from psychiatric disorders, including bipolar disorder or schizophrenia, along with higher risks of suicide [37,38].

### 4.3. Cingulate Gyrus

The connectivity of white matter in the cingulate gyrus also decreased significantly in the SA group and D group, especially the anterior part. Previous studies illustrated that the cingulate gyrus is associated with emotional processing and memory [39,40]. An fMRI study showed that the cingulate cortex had lower activations in patients with mood and anxiety disorders than in healthy individuals [41]. Additionally, studies have shown that neurons of the dorsal anterior cingulate cortex multiplex information about action and reward, which could lead to the outcome of one’s behavior [42]. Impairment of the cingulate cortex could trigger abnormal emotional responses and cause disorders, such as depression. Another study showed that the anterior cingulate cortex could be promising in the prediction of the antidepressant response [43].

### 4.4. Precuneus/Cuneus

The precuneus is another region that demonstrated significantly lower white matter integrity in the SA and D groups. It contains three subdivisions with different functional roles, including the anterior sensorimotor-related region, central cognitive/associative-related region and posterior visual-related region [44]. The precuneus is also correlated to emotion. Previous studies have shown that the precuneus is correlated with depression as well as other psychiatric disorders, including bipolar disorder and schizophrenia [45,46,47]. The precuneus, which is positioned between the cingulate and splenial sulci, is associated with behavioral functions [48]. Additionally, the precuneus is correlated with happiness [49]. For the cuneus, we found that it had a lower value of NQA in the SA group than in the D group and a lower value of GFA in the D group than in the HC group. According to previous research, the cuneus is also correlated with depression and anxiety [50]. An fMRI study found that veterans with anger and aggression problems had stronger activation in the cuneus than healthy controls [51]. These findings have provided adequate evidence that impairment of the precuneus and cuneus could lead to depression.

### 4.5. Caudate

In the comparison of the caudate, our results showed that both the SA and D groups had decreased white matter compactness compared with the HC group, while there was no significant difference in white matter connectivity or compactness between the SA group and the D group. The caudate is correlated to emotion [52]. Resting-state fMRI research showed hypoactivity in the caudate of MDD patients compared to healthy controls, while diffusion MRI research illustrated that diffusion properties in the caudate of the animals under chronic mild stress exposure were different from those in the control group [53,54]. Another pharmacological treatment study showed that ketamine could increase global signal regression (GBCr) values in the caudate of patients with major depression [55].

### 4.6. Network Measurement

Previous studies have illustrated that depression may alter the brain network [56,57]. Our results showed that both the D and HC groups had similar network tendencies without significant differences among the six topological parameters, including the global efficiency, normalized shortest path length (λ), characteristic path length, normalized clustering coefficient (γ), modularity and small worldness index in the graph theoretical analysis. For the SA group, these six topological parameters had notable differences compared to both the D and HC groups. First, the small-worldness index is the ratio of the normalized clustering coefficient (γ) to the normalized shortest path length (λ). Our analyses showed that the small-worldness index of all three groups was greater than one, which illustrated that the small-world property remained in each group. For the other five parameters, the SA group had a significantly lower global efficiency that represents the reduction of global integration. The result also showed a longer normalized shortest path length (λ) and characteristic path length, as the normalized shortest path length (λ) is the normalization of the normalized path length of 100 random brains, while the characteristic path length shows the ability to construct transitions between brain regions globally. As both values increase, global integration worsens. Furthermore, the normalized clustering coefficient (γ) is the normalization of the clustering coefficient, which quantifies interconnections between networks locally, and the modularity is the ability of the connection of nodes within local networks. However, another previous DTI study found that veterans with suicide history had greater global efficiency and shorter characteristic path lengths, which was contrary to our discoveries [58]. Our study results demonstrated an increasing normalized clustering coefficient and modularity of the SA group, which indicates that the SA group had better local segregation.

Regular networks are illustrated as networks with nodes connected to the nearest neighbors that the brain can maintain with less energy. Although regular networks display high local segregation, they remain less efficient. Random networks are the opposite of regular networks, in which two given nodes are connected by one edge. In contrast, random networks present high global integration. However, the brain is proposed to work economically [59], which makes random networks also less efficient because they have to spend as much energy to maintain brain function as the brain. Regular networks that lack global integration may be associated with lower verbal memory scores [60]. Small-world properties describe complex networks that combine random and regular networks altogether, which include both high clustering and efficiency [59].

Combining our results above, although the result of the SA group maintained the small-worldness brain network (index values > 1), it was prone to a regular network due to the increase in local segregation and the decline of global integration based on the result of our study. Our NBS analysis results showed that the SA and D groups both showed a few connections between brain regions that were more notable than the HC group, especially the frontal and parietal lobes. Previous studies have demonstrated that frontothalamic loops were abnormal in depressed patients with suicide history [61].

In the voxel-based statistical analysis, we found that some brain regions demonstrated significant impairment of white matter connectivity. To correlate our network measurement results with structural analyses, we conjectured that white matter integrity deficiency might concomitantly induce a compensatory mechanism and lead to hyperactivity of the local network, which might verify that neural basis structural changes in the brain in depressed patients with a history of suicide attempts may induce network alterations.

### 4.7. Limitations

Our study had some potential limitations. First, our participants were all Asians; therefore, it is unclear whether our results could represent the universal phenomenon. Additionally, our study only included adult participants; therefore, the findings may not generalize to juveniles. Moreover, our participants in the SA and HC groups were mostly female, which may cause gender bias. Although studies and statistical results have shown that the rates of depression are universally higher in females [62], future studies could recruit more male participants to balance the numbers.

### 4.8. Future Directions

We suggest that future studies include larger sample sizes or different clinical populations and use various kinds of imaging methods, which could offer more reliable and robust findings [63]. Future studies could also examine more evasive features of suicidal risks, including suicidal ideation with implicated psychological characteristics, including rumination and hopelessness [64,65], which could be helpful for clinical diagnoses. The brain–behavior correlations are also an important topic for future studies, since they can explore the associations between human behaviors and the brain. Additionally, different analytic techniques could be utilized to classify or predict the potential risks of suicide [66,67].

## 5. Conclusions

Accumulated evidence has shown that white matter abnormalities and network alterations are related to patients with depression as well as suicide attempts. In our study, we analyzed both neurologically structural variations and network differences in healthy controls and depressed patients with and without a history of suicide attempts using a GQI approach that improved the accuracy of fiber tracking and graph theoretical analysis. Structural abnormality in the corpus callosum, cingulate, precuneus, cuneus, caudate and network changes were shown and adequately consistent with previous studies. More research can be conducted in the future to obtain a further understanding.

## Figures and Tables

**Table 1:** Demographic characteristics.

Characteristics	SA (*n* = 44)		D (*n* = 56)		HC (*n* = 55)		ANCOVA	SA vs. HC	SA vs. D	D vs. HC
	Mean	SD	Mean	SD	Mean	SD	*p*-Value			
Age	41.32	9.37	45.48	10.53	39.4	10.70	0.008	0.344	0.040	0.003
Range of age	20–57	N/A	20–60	N/A	20–57	N/A	N/A	N/A	N/A	N/A
Gender (M/F)	7/37	N/A	24/32	N/A	9/46	N/A	N/A	0.952	0.003	0.002
Years of education	11.86	2.41	13.25	2.82	14.25	2.98	<0.001	<0.001	0.009	0.073
HAM-D	18.25	7.73	14.89	6.54	3.93	5.51	<0.001	<0.001	0.024	<0.001
HAM-D (without suicidal factor)	16.34	7.20	13.34	6.75	3.82	5.31	<0.001	<0.001	0.036	<0.001
Anxiety of HADS	11.84	5.34	7.95	4.46	4.31	3.63	<0.001	<0.001	<0.001	<0.001
Depression of HADS	11.72	4.46	7.14	4.57	3.30	3.22	<0.001	<0.001	<0.001	<0.001

Abbreviation: SA: Depressed patients with a history of suicide attempts. D: Depressed patients without a history of suicide attempts. HC: Healthy controls. HAM-D: Hamilton Rating Scale for Depression. HADS: Hospital Anxiety and Depression Scale. SD: Standard deviation.

## Data Availability

Due to the ethical approval and requirements of the data protection legislation, the data set will only be made available on a restricted basis according to the data sharing policies at the Chang Gung Memorial Hospital, Chiayi, Taiwan. Applications for access to anonymized data can be obtained by sending an e-mail to jcweng@mail.cgu.edu.tw.
